# Design of Residual Stress-Balanced Transferable Encapsulation Platform Using Urethane-Based Polymer Superstrate for Reliable Wearable Electronics

**DOI:** 10.3390/polym17192688

**Published:** 2025-10-04

**Authors:** Sung-Hun Jo, Donghwan Kim, Chaewon Park, Eun Gyo Jeong

**Affiliations:** 1 Division of System Semiconductor, Dongguk University, Seoul 04620, Republic of Korea; 2 Department of Electronics Engineering, Incheon National University (INU), 119 Academy-ro, Yeonsu-gu, Incheon 22012, Republic of Korea

**Keywords:** polymer superstrate, residual stress balancing, transferable encapsulation, ALD nano-stratified barrier, OLED reliability, iCVD

## Abstract

Wearable and skin-mounted electronics demand encapsulation designs that simultaneously provide strong barrier performance, mechanical reliability, and transferability under ultrathin conditions. In this study, a residual stress-balanced transferable encapsulation platform was developed by integrating a urethane-based copolymer superstrate [p(IEM-co-HEMA)] with inorganic thin films. The polymer, deposited via initiated chemical vapor deposition (iCVD), offered over 90% optical transmittance, low RMS roughness (1–3 nm), and excellent solvent resistance, providing a stable base for inorganic barrier integration. An ALD Al_2_O_3_/ZnO nano-stratified barrier initially delivered effective moisture blocking, but tensile stress accumulation imposed a critical thickness of 30 nm, where the WVTR plateaued at ~2.5 × 10^−4^ g/m^2^/day. To overcome this limitation, a 40 nm e-beam SiO_2_ capping layer was added, introducing compressive stress via atomic peening and stabilizing Al_2_O_3_ interfaces through Si–O–Al bonding. This stress-balanced design doubled the critical thickness to 60 nm and reduced the WVTR to 3.75 × 10^−5^ g/m^2^/day, representing an order-of-magnitude improvement. OLEDs fabricated on this ultrathin platform preserved J–V–L characteristics and efficiency (~4.5–5.0 cd/A) after water-assisted transfer and on-skin deformation, while maintaining LT80 lifetimes of 140–190 h at 400 cd/m^2^ and stable emission for over 20 days in ambient storage. These results demonstrate that the stress-balanced encapsulation platform provides a practical route to meet the durability and reliability requirements of next-generation wearable optoelectronic devices.

## 1. Introduction

Wearable electronics, skin-mounted devices, and flexible optoelectronic systems have rapidly evolved into essential technologies for applications such as real-time health monitoring, interactive human–machine interfaces, and immersive display experiences [1,2,3,4]. These devices often operate on curved, deformable, or irregular surfaces, including human skin and textiles, where long-term operational stability must be maintained under mechanical deformation and environmental exposure. Achieving such performance requires a supporting layer that is not only mechanically compliant but also exceptionally thin, lightweight, and capable of maintaining structural integrity during processing and use.

Mechanical compliance is one of the most critical parameters in designing platforms for wearable and conformable electronics. The bending stiffness of a film depends on both the effective elastic modulus and, more significantly, the cube of its thickness [5,6]. For example, reducing thickness from 50 µm to 5 µm decreases the bending stiffness by roughly three orders of magnitude, so few-micrometer platforms are typically regarded as ultrathin for wearable applications. Thus, reducing thickness into this range yields dramatic improvements in conformability. However, such thinning also comes with challenges, particularly limited barrier performance and increased susceptibility to residual stress.

Commercial plastic films such as polyimide (PI), polyethylene terephthalate (PET), and polyethylene naphthalate (PEN) are typically manufactured by melt extrusion, solution casting, or stretching processes, and their available thickness is generally in the tens of micrometers. Achieving free-standing films in the few-micrometer range often requires carrier or release processes, which introduce handling and yield challenges and limit their direct applicability to ultrathin, skin-mounted systems. In addition, these materials possess limited surface functionalities, so additional treatments such as plasma activation are often required to ensure continuous and uniform deposition.

In contrast, the initiated chemical vapor deposition (iCVD) process enables solvent-free, low-temperature deposition of polymer films with precisely controlled thickness, high optical transparency, low RMS roughness (≈1–3 nm), and excellent solvent resistance [7,8,9,10]. More importantly, functional groups can be tailored within the polymer to enhance interfacial affinity with subsequently deposited inorganic layers, enabling dense and continuous growth. However, as with other polymers, the intrinsic water vapor transmission rates (WVTR) of the iCVD film alone is insufficient (10^−1^–10^2^ g/m^2^/day), which is far from the stringent requirement of 10^−6^ g/m^2^/day for organic light-emitting diodes (OLEDs) and organic photovoltaics (OPVs) [11]. In industrial practice, a WVTR of approximately 10^−5^ g/m^2^/day under 40 °C/90% RH with processing temperatures below 100 °C, is typically regarded as the module standard [12]. Accordingly, multilayer encapsulation strategies continue to be developed to bridge this gap [13,14,15,16].

For wearable electronics, recent studies have reported biocompatible multilayer encapsulation structures designed to ensure reliability even in aqueous or biological environments [17]. However, their total thickness often reaches several micrometers, limiting conformability for skin-mounted ultrathin platforms. To address this, hybrid polymer–inorganic thin films have been proposed, combining the flexibility of polymers with the barrier capability of inorganics, yet their WVTR performance remains limited [18]. To overcome the inherently slow growth rate of atomic layer deposition (ALD), spatial ALD has also been introduced, enabling high-throughput, large-area deposition. Nevertheless, despite improvements in throughput, stress accumulation and mechanical durability remain unresolved challenges [19].

These limitations fundamentally arise from the trade-off between WVTR improvement and residual stress accumulation in inorganic barriers. As oxide thickness increases, barrier performance initially improves, but the accumulated tensile stress eventually induces microcracks that stagnate or even worsen WVTR. This issue becomes particularly critical on ultrathin platforms, where even small amounts of stress can trigger severe deformation or delamination [20,21,22,23]. To mitigate this, wrinkle or wavy surface structures have recently been introduced to relax internal stress and delay crack formation [24,25]. However, these morphology-driven approaches are strongly dependent on substrate conditions, and their reliability has not been directly validated at the device level, such as after transfer or during on-skin operation.

To overcome these limitations, this study introduces a stress-balanced transferable encapsulation platform. The platform employs a urethane-based copolymer, p(IEM-co-HEMA), deposited by iCVD serving as a stable superstrate for inorganic layer. On top of this, an ALD Al_2_O_3_/ZnO nano-stratified encapsulation barrier was integrated [26]. Amorphous Al_2_O_3_ provides superior moisture-blocking capability, while ZnO acts as a crack arrester at the interfaces during deposition [27]. This combination produces a synergistic effect that mitigates the trade-off between barrier performance and mechanical flexibility. However, as the thickness increases, tensile residual stress accumulates, leading to a WVTR plateau that defines the critical thickness (30 nm) of the TFE. To address this, an e-beam-deposited SiO_2_ capping layer was added, introducing compressive stress through atomic peening and enhancing interfacial stability via the formation of Si–O–Al bonds.

As a result, the critical thickness of the ALD encapsulation barrier was extended to 60 nm, and the WVTR was improved by approximately one order of magnitude, from 2.47 × 10^−4^ to 3.75 × 10^−5^ g/m^2^/day under identical conditions. More importantly, OLEDs fabricated on this platform exhibited no additional electrical or optical losses during the water-assisted transfer process and maintained stable shelf-lifetime for more than 20 days after on-skin deformation tests. These findings verify the effectiveness of the proposed stress-balanced design at both barrier and device levels, demonstrating that the ultrathin transferable platform not only improves barrier performance but also ensures reliability under practical wearable operating conditions. Therefore, the stress-balanced strategy presented in this work offers a viable pathway to meet the stringent requirements of next-generation skin-mounted electronic devices.

## 2. Materials and Methods

### 2.1. Fabrication of p(IEM-co-HEMA) Superstrate

p(IEM-co-HEMA) films with a molar feed ratio of 1:1 (pI1H1) were deposited via iCVD onto 4-inch silicon wafers pre-coated with a 30 nm poly(vinyl alcohol-co-itaconic acid) (pVI, Sigma-Aldrich) sacrificial layer. Isocyanatoethyl methacrylate (IEM, Sigma-Aldrich, 98%) and 2-hydroxyethyl methacrylate (HEMA, TCI Chemicals, 95%) monomers were vaporized and introduced into the chamber together with tert-butyl peroxide (TBPO, Sigma-Aldrich, 98%) initiator delivered at a constant flow rate of 0.6 sccm. The chamber pressure was fixed at 200 mTorr, and the filament array was heated to 140 °C to thermally decompose TBPO and generate free radicals, initiating polymerization directly on the substrate surface. Film thickness was controlled by deposition time at a rate of ~20 nm/min and monitored by interferometry.

### 2.2. Deposition of ALD Nano-Stratified Barrier

Al_2_O_3_ and ZnO sublayers were deposited via thermal ALD at a chamber temperature of 70 °C to construct nano-stratified barrier structures. Al_2_O_3_ sublayers were deposited using trimethylaluminum (TMA, 99.999% trace metals basis) as the aluminum precursor and deionized water (H_2_O) as the reactant, whereas ZnO sublayers were deposited using diethylzinc (DEZ, 95%) as the zinc precursor with H_2_O as the reactant. Each ALD cycle consisted of a 0.2 s precursor pulse (TMA or DEZ) to saturate the substrate surface with the respective metal-containing species, followed by a 10 s high-purity nitrogen (N_2_, 99.9999%) purge to remove unreacted precursor molecules and by-products from the reaction chamber. Subsequently, a 0.2 s H_2_O pulse was introduced to hydrolyze the chemisorbed precursor layer, forming the desired metal–oxygen bonds. A second 10 s N_2_ purge completed the cycle by removing excess reactants and gaseous by-products. This cyclic sequence ensured layer-by-layer growth with sub-nanometer thickness control. For the nano-stratified barrier, individual Al_2_O_3_ and ZnO sublayers were each deposited to a nominal thickness of 3 nm, corresponding to approximately 30 cycles for Al_2_O_3_ and 28 cycles for ZnO under the given conditions.

### 2.3. Deposition of SiO_2_ Capping Layer

SiO_2_ capping layers were deposited using three different physical vapor deposition (PVD) methods—thermal evaporation, electron-beam (E-beam) evaporation, and sputtering—to evaluate the effect of deposition method on residual stress and barrier performance. For thermal evaporation, high-purity SiO_2_ pellets were resistively heated under a base pressure of 1 × 10^−6^ Torr, with a deposition rate of 1.0–1.2 Å/s. For e-beam evaporation, the same SiO_2_ source was irradiated with a focused electron beam under identical base pressure conditions, with a deposition rate of 1.0–1.5 Å/s. For sputtering, a SiO_2_ target (99.99%, 3-inch diameter) was sputtered in an Ar atmosphere (30 sccm, 3 mTorr) at an RF power of 100 W, yielding a deposition rate of approximately 0.8 Å/s. In all cases, the target thickness of the SiO_2_ capping layer was fixed at 40 nm, corresponding to a deposition time of approximately 5–7 min depending on the method.

### 2.4. Fabrication of Stress-Balanced Transferable Platform

The stress-balanced transferable encapsulation platform was fabricated in a multilayer configuration to ensure both mechanical reliability and chemical stability during water-assisted delamination. First, a 40 nm SiO_2_ capping layer was deposited onto the p(IEM-co-HEMA) superstrate via e-beam evaporation. Subsequently, a 60 nm ALD nano-stratified barrier was deposited at 70 °C. Top-emission OLED devices were then fabricated on the barrier-coated polymer superstrates with the following structure: aluminum (Al, 100 nm, cathode)/8-hydroxyquinolinolato-lithium (Liq, 1 nm, electron-injection layer)/tris(8-hydroxyquinolinato)aluminum (Alq_3_, 50 nm, emissive layer)/N,N′-bis(1-naphthyl)-N,N′-diphenyl-1,1′-biphenyl-4,4′-diamine (NPB, 50 nm, hole-transport layer)/molybdenum trioxide (MoO_3_, 5 nm, hole-injection layer)/silver (Ag, 30 nm, anode)/NPB (50 nm, optical capping layer). All organic and metal layers were deposited by thermal evaporation under a base pressure of 1 × 10^−6^ Torr. Following OLED deposition, a second 60 nm ALD nano-stratified barrier and a final 40 nm SiO_2_ capping layer were sequentially deposited to complete the encapsulation. Finally, the entire device stack (Section 3.4) was exfoliated from the silicon carrier wafer by immersing it in deionized water, which dissolved the underlying pVI sacrificial layer, yielding a free-standing, transferable platform suitable for integration onto arbitrary target surfaces.

### 2.5. Characterization

The surface morphology and root mean square roughness (R_q_) were measured using an atomic force microscope (AFM, XE-100, Park Systems, Suwon, Republic of Korea) in non-contact mode to avoid tip-induced damage. Measurements were performed over a scan area of 5 × 5 μm^2^, with height data recorded at a resolution of 512 × 512 pixels. Samples were mounted directly onto the AFM stage without additional conductive coating to preserve native surface conditions. Rq values were determined using the built-in analysis software, and the results were averaged over at least three different regions per sample to ensure reproducibility.

Optical transmittance was measured using a UV–Vis spectrophotometer (Shimadzu UV-2550, Kyoto, Japan) over a wavelength range of 300–800 nm with a spectral resolution of 1 nm. Measurements were performed on films deposited on quartz substrates to eliminate background absorption from the support. Refractive index values were obtained by spectroscopic ellipsometry (M-2000, J.A. Woollam Co., Lincoln, NE, USA) over the 300–1000 nm wavelength range, and data were fitted using a Cauchy dispersion model. Film thickness was simultaneously determined during ellipsometry fitting, and these values were cross-validated with profilometry measurements.

Thermal stability of film was evaluated using thermogravimetric analysis (TGA/DSC 3+, Mettler-Toledo, Greifensee, Switzerland). Samples were heated from room temperature to 600 °C at a rate of 10 °C/min under a nitrogen atmosphere (50 mL/min) to determine the onset decomposition temperature. The coefficient of thermal expansion (CTE) was measured using thermomechanical analysis (TMA Q400, TA Instruments, New Castle, DE, USA) in film tension mode over the temperature range of 25–200 °C at a heating rate of 5 °C/min. Film strips (5 × 20 mm^2^) were prepared from the same deposition batch to ensure consistency.

The elastic modulus and Poisson’s ratio of both the polymer and ALD-deposited inorganic films were evaluated using a nanoindenter (Nano Indenter XP, MTS, Eden Prairie, MN, USA) equipped with a Berkovich diamond tip. Indentation depths were kept within the film thickness to avoid substrate influence, and the extracted values were employed for residual stress analysis. Residual stress of the ALD barrier films was further determined using a wafer curvature method with an FSM-500tc system (FSM, San Jose, CA, USA). Barrier structures were deposited on silicon wafers, and the wafer curvature was recorded before and after deposition. Residual stress was then calculated from the curvature change using Stoney’s equation, incorporating the measured elastic modulus, Poisson’s ratio, and thickness values of both the substrate and the film.

The electrical calcium test was used to quantify the WVTR of barrier-coated superstrates. A 100 nm thick aluminum electrode was thermally evaporated onto a glass substrate, followed by deposition of a 250 nm calcium pad (1.5 cm^2^ active area) by thermal evaporation at 1 × 10^−6^ Torr. The calcium pad was sealed with the barrier-coated p(IEM-co-HEMA) superstrate using a UV-curable sealant (XNR 5570-Ba, Nagase Chemtex, Osaka, Japan) inside an N_2_-filled glovebox to avoid premature reaction with ambient moisture. After sealing, the samples were transferred to a temperature–humidity chamber maintained at 30 °C and 90% relative humidity. The resistance of the calcium pads was continuously recorded in real time using a sheet resistance meter (Keithley 2750, Cleveland, OH, USA), enabling WVTR evaluation under accelerated environmental conditions. The WVTR values were determined from the calcium degradation rate induced by permeating water vapor and oxygen [28].

Fourier-transform infrared (FT-IR) spectroscopy in attenuated total reflection (ATR) mode (IFS 66 V/S & Hyperion 3000, Bruker, Ettlingen, Germany) was used to examine the chemical bonding states of barrier films before and after moisture exposure. Spectra were collected in the range of 4000–600 cm^−1^ with a resolution of 4 cm^−1^, averaging 32 scans per measurement. Samples were analyzed immediately after deposition and after dipping tests to assess chemical stability.

Electroluminescent performance of fabricated OLEDs was characterized by measuring current density–voltage–luminance (J–V–L) characteristics using a source meter (Keithley 2400, Tektronix, Cleveland, OH, USA) and a spectroradiometer (CS-2000, Konica Minolta, Tokyo, Japan) under ambient conditions. Current efficiency–current density curves were also obtained to evaluate device efficiency. For these measurements, the voltage was swept from 0 to 8.5 V in 0.5 V steps using the source meter, while the luminance was simultaneously recorded by the spectroradiometer. The current efficiency was calculated as the ratio of luminance to current density. Measurements were conducted both before and after water-assisted delamination to assess the impact of the transfer process on device performance.

## 3. Results & Discussion

### 3.1. Urethane-Based Polymer Superstrate for Conformable and Transferable Platforms

Wearable electronic devices, particularly those operating in direct contact with skin or textile surfaces such as electronic skin, require substrates with both high conformability and compatibility with transfer process. High conformability necessitates low bending stiffness, while minimizing mechanical damage during transfer requires low surface roughness and residual stress. Since bending stiffness is governed by both the effective elastic modulus and, more dominantly, the cube of the thickness, suppressing the thickness to the range of several hundred nanometers to a few micrometers is the most direct and effective design parameter. Reducing bending stiffness not only improves mechanical compliance but also allows the substrate to conform to irregular or curved surfaces without inducing localized strain concentrations that can damage active device layers. In addition, controlling residual stress is critical for ensuring intimate contact between layers and maintaining structural integrity during repeated deformation or environmental exposure.

**Figure 1:**
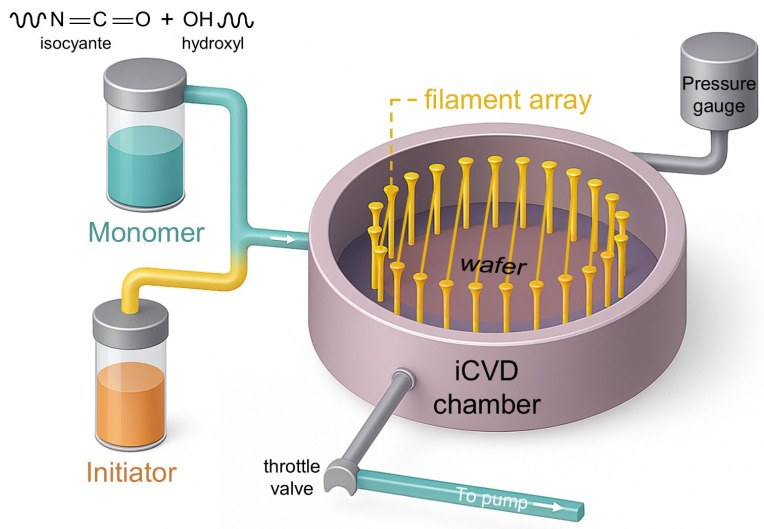
Schematic illustration of the iCVD process for fabricating a p(IEM-co-HEMA) superstrate.

To meet these requirements, a urethane-group-based polymer film—p(IEM-co-HEMA)—was fabricated via iCVD, as illustrated in Figure 1. In this process, monomers are supplied in the vapor phase, and polymerization is initiated directly on the substrate surface using a heated filament array as the radical initiator source. The solvent-free and low-temperature nature of iCVD minimizes issues such as residual solvent contamination, swelling of underlying layers, and thermal damage to temperature-sensitive substrates, which are common in conventional solution-based processes. In addition, iCVD inherently provides excellent film uniformity over large substrate areas with minimal defects and thickness variation. Furthermore, the surface-reaction-limited growth mechanism of iCVD allows precise and continuous control of film thickness, enabling fine adjustment of mechanical flexibility and process compatibility for transfer processes.

The resulting p(IEM-co-HEMA) superstrate combines extreme thinness with biocompatibility, making it suitable as a transferable support layer for wearable electronics. Its measured physical, optical, chemical, and mechanical properties are summarized in Table 1. The film exhibits sub-3 nm surface roughness, which promotes intimate contact with overlying functional layers and minimizes interfacial defects. Optical transmittance exceeding 90% across the visible range allows its use in optically active devices without significant light loss. The polymer exhibits excellent resistance to water, alcohols, and common organic solvents, with no noticeable dimensional changes during processing. Mechanically, it maintains an elastic modulus of 1–3 GPa despite its microscale thickness, ensuring sufficient mechanical stability during device fabrication while remaining compliant enough to conform to non-planar surfaces during transfer. These combined properties guarantee minimal optical or structural interference when integrated with active device layers, thereby expanding the range of potential applications from flexible displays to skin-mounted sensor systems.

### 3.2. Correlation Between Residual Stress and WVTR in ALD Nano-Stratified Barriers

The p(IEM-co-HEMA) superstrate provides excellent conformability and transferability, making it suitable for integration into flexible and transferable electronic devices. However, its intrinsic barrier capability is insufficient to ensure the long-term stability of devices highly sensitive to moisture and oxygen ingress. For flexible organic optoelectronic devices, operational stability often requires a WVTR below 10^−5^ g m^−2^ day^−1^, a specification that the polymer superstrate alone cannot meet. To address this limitation, an inorganic thin-film encapsulation barrier was introduced using thermal ALD. ALD, based on self-limiting surface reactions, enables sub-nanometer thickness control, large-area uniformity, and the formation of dense, pinhole-free films. Unlike general PVD methods, ALD is not constrained by a line-of-sight requirement, enabling uniform coating even on high-aspect-ratio structures and complex geometries. This capability is a critical advantage for minimizing defect formation in barrier layers.

**Figure 2:**
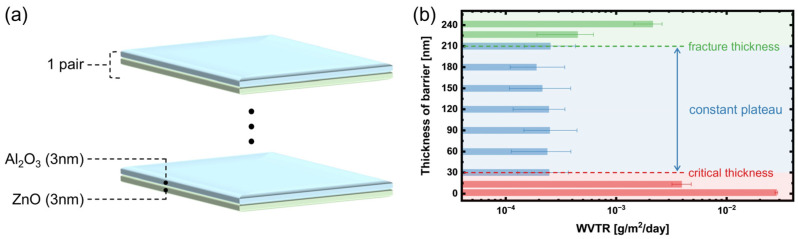
ALD nano-stratified Al_2_O_3_/ZnO barrier. (**a**) Schematic illustration of alternating Al_2_O_3_/ZnO sublayers. (**b**) WVTR as a function of barrier thickness, indicating the critical thickness, the plateau regime, and fracture thickness.

To exploit the complementary advantages of Al_2_O_3_ as a dense amorphous moisture barrier and ZnO as a crack arrester, a nano-stratified structure of alternating 3 nm sublayers was adopted. The sublayer thickness was selected to match the surface roughness of the p(IEM-co-HEMA) film, ensuring conformal coverage of surface features. This configuration formed a nano-stratified structure, as illustrated in Figure 2a. The alternating sequence increases the tortuosity of the diffusion path for water vapor and oxygen molecules, reducing the likelihood of continuous defect channels compared to a single-layer barrier. As shown in Figure 2b, WVTR decreases sharply with the initial increase in thickness due to barrier densification and elimination of defect-mediated permeation pathways. Beyond 5 pairs (30 nm), WVTR reaches a constant plateau, defined as the critical thickness, where additional layers no longer produce measurable performance gains [29,30,31]. However, when the total barrier thickness further increases and exceeds 35 pairs (210 nm), WVTR begins to rise again. This behavior corresponds to the fracture thickness, which has been defined as the thickness limit beyond which residual stress accumulation in the film leads to the formation of cracks and new permeation pathways.

**Figure 3:**
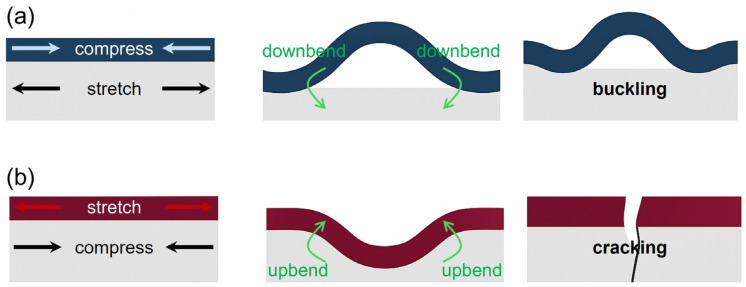
Schematic illustrations of failure mechanisms in thin films under different residual stress states. (**a**) Compressive residual stress induces buckling. (**b**) Tensile residual stress leads to cracking.

The increase in WVTR beyond the fracture thickness is attributed to residual stress generated during film growth, as illustrated in Figure 3. Residual stress originates from chemical reactions, atomic diffusion, and thermal expansion mismatch between sublayers during deposition, and it directly affects the mechanical stability and long-term barrier performance. In the case of compressive residual stress, the barrier layer bends downward toward the substrate, creating an in-plane compressive state. While mild compression can help suppress the opening of microcracks and thus be beneficial in the short term, excessive compression causes local buckling, disrupting the planar continuity of the film. If interfacial adhesion is insufficient, partial delamination may occur at the barrier–substrate interface. These buckled or delaminated regions, even if not visible on the surface, interrupt the intentionally tortuous diffusion pathway of the encapsulation barrier architecture, creating localized sites where moisture and oxygen permeation is accelerated.

Conversely, tensile residual stress stretches the barrier layer (Figure 3b), causing upward bending and concentrating strain energy at defects such as pinholes or grain boundaries. With continued stress accumulation, cracks form within the film, creating permeation pathways and leading to the sharp rise in WVTR observed beyond the fracture thickness. In polymer substrates with relatively low bending stiffness, the barrier becomes even more susceptible to such cracking. Consequently, barrier designs that remain mechanically stable on rigid substrates may lose stability and crack much more rapidly when applied to flexible, transferable polymer-based platforms.

Therefore, as the thickness of the superstrate decreases, the influence of residual stress must be considered more carefully. According to Stoney’s equation (Equation (1)) [32], thinner superstrates exhibit larger curvature changes under the same residual stress conditions:

(1) $$\sigma =\frac{E_{s}t_{s}^{2}}{6\left(1-\nu_{s}\right)t_{f}R}$$

where *σ* is the average residual stress in the film, *E_s_* is the Young’s modulus of the substrate, *ν_s_* is the Poisson’s ratio of the substrate, *t_s_* is the substrate thickness, *t_f_* is the film thickness, and *R* is the radius of curvature of the bent substrate–film system. Hence, when the substrate is thinner and more compliant, *R* decreases, and the same residual stress produces much larger curvature. In ultrathin polymer superstrates, this geometric effect amplifies the curvature response, increasing the likelihood of microcrack formation or delamination within the barrier layer.

**Figure 4:**
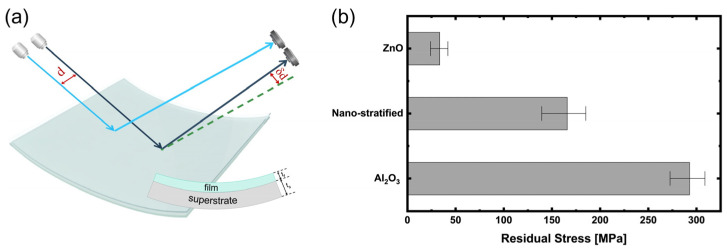
Residual stress in ALD-based barrier films. (**a**) Schematic illustration of residual stress measurement using a laser scanning method. (**b**) Residual stress comparison among single-layer Al_2_O_3_, single-layer ZnO, and nano-stratified (Al_2_O_3_/ZnO) structures.

Residual stress of ALD-based inorganic thin-film barriers was analyzed using the laser scanning method shown in Figure 4a. Thin-film barriers identical to those used on polymer superstrates were deposited on Si wafers, and the change in spot spacing (δd) of reflected laser beams before and after deposition was measured to determine wafer curvature. The residual stress was then calculated using Stoney’s equation. Although Si and polymer substrates differ in modulus, the intrinsic stress of the ALD films is process-determined and therefore comparable. Furthermore, the CTE of Al_2_O_3_, ZnO, and Si are similar (4.2, 4.7, and 3.0 ppm/K, respectively), so thermal mismatch stress is negligible and the measured values mainly reflect intrinsic growth stress. As shown in Figure 4b, Al_2_O_3_ exhibited the highest tensile residual stress (~290 MPa), ZnO showed a low stress level (~30 MPa).

Since thermal ALD Al_2_O_3_ film grows in an amorphous structure, the removal of –CH_3_ groups and the formation of Al–O–Al crosslinks cause cumulative micro-scale volume shrinkage and densification. However, due to its amorphous nature, there are no stress-relief pathways such as grain boundary sliding or dislocation motion, leading to the accumulation of high tensile residual stress. In contrast, ZnO deposited by ALD grows in a polycrystalline wurtzite structure, where the presence of grain boundaries provides structural pathways for partial stress relaxation, resulting in lower residual stress compared to amorphous Al_2_O_3_. In the nano-stratified structure, however, the ZnO sublayer thickness is restricted to only a few nanometers, limiting crystal growth and preventing full activation of these relaxation mechanisms. As a result, the total tensile residual stress of the nano-stratified layer remains lower than that of single-layer Al_2_O_3_ but higher than that of ZnO, at approximately 170 MPa.

These results demonstrate that the nano-stratified structure is an effective design for achieving both excellent WVTR performance and a certain degree of stress mitigation. However, a non-negligible level of residual stress remains, and exceeding the critical thickness inevitably results in stress-induced cracking and degradation of barrier performance. Furthermore, under the current critical thickness, the WVTR has not yet reached a sufficiently low level. Therefore, design and process strategies that optimize residual stress to extend the critical thickness are essential for achieving improved barrier performance. Such optimization is crucial for ensuring both the long-term stability and the mechanical reliability of the barrier layer.

### 3.3. Stress Balancing and Chemical Stabilization Strategy via E-Beam SiO_2_ Layer

Beyond the critical thickness, the accumulation of tensile residual stress induces cracks in the film, leading to stagnation or deterioration of barrier performance. Unlike in conventional fracture mechanics, where cracks must grow to a critical crack length before causing failure, in TFE even smaller cracks can act as diffusion pathways for moisture and oxygen, thereby degrading WVTR. One effective approach to suppress this phenomenon is to intentionally introduce compressive residual stress during the growth stage, thereby balancing the overall residual stress. Representative methods include shot peening, cold rolling, and stretching, which apply in-plane compressive deformation to the surface, increasing resistance to crack propagation and extending fatigue life [33,34].

**Figure 5:**
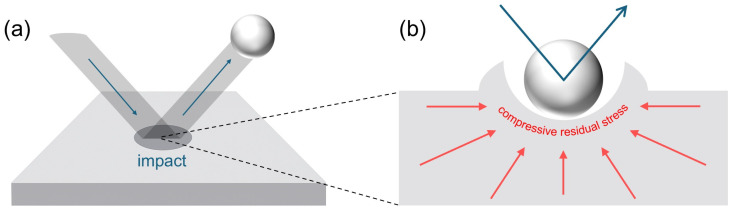
Schematic illustration of atomic peening during deposition. (**a**) High-energy particle bombardment on the film surface. (**b**) Enlarged view showing the generation of compressive residual stress beneath the impact site.

When this principle is applied to thin-film growth processes, high-energy particles or ions bombard the growing film surface, as illustrated in Figure 5, leading to an atomic peening effect where surface atoms are driven into subsurface layers or densified [35]. This suppresses free in-plane expansion and induces compressive residual stress, with the effect being particularly pronounced in PVD processes. Key parameters such as ion energy, incidence angle, and flux determine the magnitude and distribution of the compressive residual stress. Additionally, localized thermal spikes generated by ion bombardment enhance the mobility of implanted atoms, facilitating atomic rearrangement and partially relieving intrinsic tensile stress.

However, before implementing a PVD-based compressive stress control layer, it must be considered that the superstrate delaminates through dissolution of the underlying sacrificial pVI layer. In this case, the p(IEM-co-HEMA) polymer layer itself remains chemically and mechanically stable even under prolonged water exposure. However, the ALD layer employed as the encapsulation barrier is susceptible to hydrolysis of Al–O–Al bonds upon long-term contact with moisture, leading to the formation of Al–OH groups, as shown in Equation (2) [36]:

(2) $$\equiv Al-O-Al\equiv +H_{2}O\to \equiv Al-OH+HO-Al\equiv$$

The generated hydroxyl (-OH) groups increase surface hydrophilicity, causing repeated swelling and shrinkage during hydration–dehydration cycles. These structural changes destabilize the internal stress distribution of the barrier and locally promote crystallization of the amorphous Al_2_O_3_ [26]. The resulting grain boundaries act as rapid diffusion pathways for water and oxygen, leading to a marked decline in barrier performance.

Therefore, selecting a capping material that ensures both residual stress balancing and long-term moisture stability is critical. From this perspective, SiO_2_ is a compelling choice, as the surface –Si–OH groups react with –Al–OH groups on Al_2_O_3_ via protonation–deprotonation to form covalent Si–O–Al bonds, as shown in Equation (3): [37]

(3) $$\equiv Si-OH+\equiv Al-OH⇌\equiv Si-O-Al\equiv +H_{2}O$$

This reaction consumes free –OH groups at the interface, suppressing water-induced hydrolysis (Al–O–Al bond cleavage). The resulting stable Si–O–Al network enhances interfacial chemical stability and blocks re-entry of moisture, ensuring long-term barrier performance. Thus, SiO_2_ simultaneously provides a primary function of preventing chemical degradation during delamination and a secondary function of imparting compressive stress through atomic peening during PVD, effectively counteracting the tensile stress originating from the ALD process.

**Figure 6:**
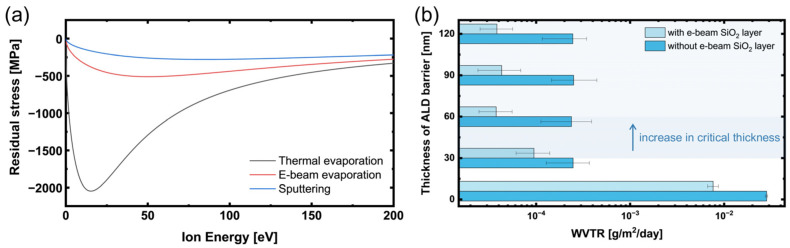
Influence of deposition method and SiO_2_ layer. (**a**) Residual stress variation with ion energy for SiO_2_ films deposited by various PVD methods. (**b**) WVTR as a function of ALD barrier thickness with and without the SiO_2_ layer.

The deposition method of the SiO_2_ capping layer must be carefully selected to ensure excellent capping performance and stable stress control while minimizing physical damage to the platform. To this end, three PVD-based methods—thermal evaporation, e-beam evaporation, and sputtering—were employed to deposit SiO_2_ films. The relationship between particle energy distribution and residual stress for each method was analyzed using the Davis model described in Equation (4) [38]:

(4) $$\sigma \left(E\right)\propto \frac{Y}{1-\nu }\frac{E^{1/2}}{R/j+kE^{5/3}}$$

where *E* is the incident ion energy, *Y* is the Young’s modulus of the film material, and *ν* is the Poisson’s ratio. *R* and *j* represent the deposition flux and bombardment flux, respectively, while *k* is a constant associated with the thermal spike effect. Figure 6a shows the calculated trend from the Davis model, whereas Table 2 summarizes the experimentally measured residual stresses and the corresponding ion energies estimated from the model.

At low ion energies, the knock-on implantation effect dominates. In this process, incident ions transfer momentum to surface atoms, driving them beneath the surface, densifying the film, and suppressing in-plane expansion, thereby producing compressive residual stress. As a result, compressive stress gradually increases with ion energy in this range. At higher ion energies, the thermal spike effect becomes dominant, where localized and transient heating promotes atomic rearrangement and defect annihilation, partially relieving the compressive stress generated by implantation. Consequently, beyond a certain threshold energy, this relaxation effect offsets the implantation effect, leading to a decrease in compressive stress.

Considering these effects, sputtering can impart strong compressive residual stress through high-energy ion bombardment; however, particle and ion energies on the order of several tens of eV can cause significant physical damage to the p(IEM-co-HEMA)-based polymer superstrate. Such damage may include increased surface roughness, localized thermal deformation, and irreversible chain scission of the polymer, all of which can degrade the performance. Therefore, although sputtering can be effective in inducing the atomic peening effect, the associated risk of substrate damage makes it unsuitable for this platform. Thermal evaporation poses minimal risk of substrate damage due to its extremely low particle energies, but the contribution of growth and thermal stresses is high, making the stress state sensitive to variations in film thickness and deposition temperature and lowering reproducibility. In contrast, e-beam evaporation offers a favorable compromise, with relatively low particle energy that minimizes substrate damage while effectively compensating for the tensile residual stress originating from ALD films.

As shown in Figure 6b, depositing an e-beam SiO_2_ capping layer onto a p(IEM-co-HEMA)-based polymer superstrate increased the critical thickness of the ALD nano-stratified barrier from 30 nm to 60 nm and reduced the WVTR from 2.47 × 10^−4^ to 3.75 × 10^−5^ g/m^2^/day, an improvement of about one order of magnitude. To quantify this effect, the membrane force was calculated as the sum of residual stress multiplied by layer thickness (Σσ·t) [39]. At the critical thickness of 30 nm without the capping layer, the membrane force is ~5100 MPa·nm, and with a 40 nm SiO_2_ capping it remains essentially the same (~5140 MPa·nm) even when the ALD barrier is extended to 60 nm. This correspondence indicates that the WVTR plateau coincides with a threshold membrane force, and that the SiO_2_ capping layer shifts this threshold to a larger thickness by compensating the tensile stress of the ALD barrier.

### 3.4. Realization of a Stress-Balanced and Highly Reliable Transferable Platform

**Figure 7:**
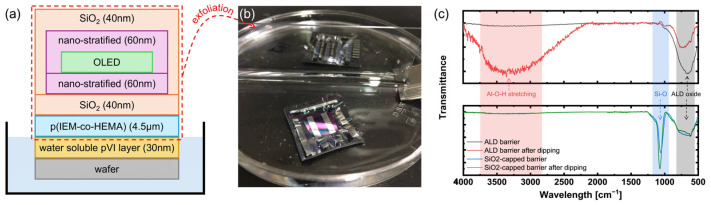
Device structure and FT-IR analysis. (**a**) Schematic of the OLED device and exfoliation process. (**b**) Photograph of water-assisted exfoliation process. (**c**) FT-IR spectra of ALD barrier and SiO_2_-capped barrier films before and after water dipping.

To achieve both stress balancing and chemical protection, the lower and upper interfaces were symmetrically designed, as shown in Figure 7a. On the superstrate side, an e-beam SiO_2_ layer was first deposited to introduce compressive residual stress, thereby effectively counteracting the tensile residual stress generated in the subsequent ALD nano-stratified barrier. The ALD nano-stratified layer serves as the primary barrier to secure inherent WVTR performance. Conversely, on the upper interface after OLED deposition, the ALD nano-stratified barrier was first formed to provide the fundamental gas-blocking capability, followed by an e-beam SiO_2_ capping layer to suppress surface chemical reactions during the water-based exfoliation process. In this configuration, the ALD layer can be protected by the SiO_2_ capping layer during delamination, thereby preventing hydrolysis of the ALD barrier. As shown in Figure 7b, the fabricated platform exhibited uniform and stable exfoliation behavior in water.

The FT-IR analysis results in Figure 7c further validate the effectiveness of this approach in maintaining chemical stability. For the ALD-only barrier without the SiO_2_ capping layer, the as-deposited sample exhibited a single absorbance peak corresponding to the characteristic frequency band of ALD oxides. However, after the dipping test, the normalized O–H stretching band near 3300 cm^−1^ [40], increased by approximately 1100%, clearly indicating hydrolysis-induced degradation of the Al_2_O_3_ layer upon moisture exposure. In contrast, the SiO_2_-capped barrier consistently exhibited two characteristic peaks both before and after the dipping test: the first peak, associated with the ALD oxide, and the second peak near 1100 cm^−1^ [37], attributed to the Si–O bond of the e-beam SiO_2_ capping layer. Importantly, in the capped sample the O–H band showed no significant change after dipping, and the Si–O peak remained clearly visible, confirming that the capping layer effectively suppresses moisture-induced hydrolysis and preserves the chemical integrity of the barrier.

**Figure 8:**
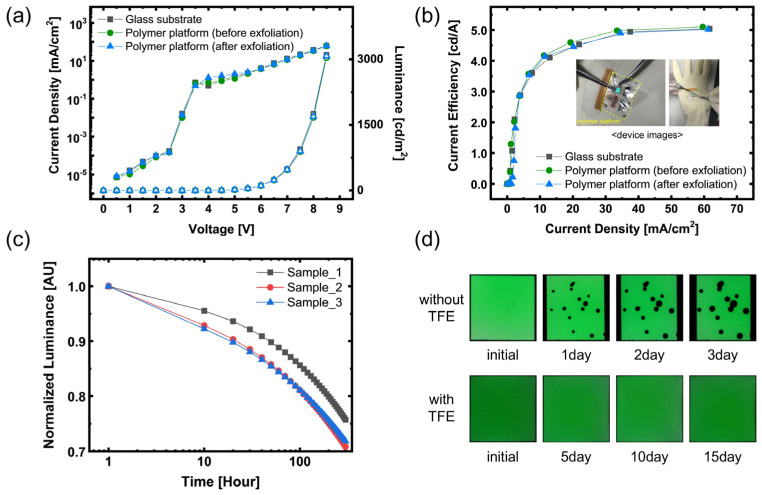
Electrical and optical performance of OLEDs on different platforms. (**a**) Current density–voltage–luminance (J–V–L) characteristics of OLEDs on a glass substrate, a polymer platform before exfoliation, and a polymer platform after exfoliation. (**b**) Current efficiency–current density curves of OLEDs on the same platforms. (**c**) Operation lifetime under constant-current driving, showing normalized luminance over time. (**d**) Photographs of OLEDs on the polymer platform and emission images with and without TFE over 20 days.

The electro-optical stability of the fabricated OLEDs was evaluated by comparing the current density–voltage–luminance (J–V–L) and current efficiency–current density characteristics between devices fabricated on the polymer platform and glass references (Figure 8a and Table 3). The polymer platform devices exhibited nearly identical turn-on voltage, slope, and maximum luminance to the glass references, and no degradation was observed even after water-assisted exfoliation. As shown in Figure 8b, the maximum efficiency (~5.0 cd/A) and the roll-off behavior also remained unchanged, confirming that the transfer process preserved charge-injection interfaces and the recombination zone. This stability can be attributed to the water-assisted exfoliation mechanism, in which the sacrificial layer dissolves without exposing the OLED to plasma, etchants, or solvents, while the TFE protects the device from water ingress.

The inset presents a device placed on a curved hand surface and subjected to natural folding, wrinkling, and local pressing; the OLED maintained uniform emission without visible cracks or delamination, which means that the ultrathin platform can withstand wearable-relevant mechanical stress. Following this on-skin deformation test, the lifetime was evaluated under constant-current driving at an initial luminance of 400 cd/m^2^ (Figure 8c). The normalized luminance decay curves yielded LT80 values of approximately 140–190 h, demonstrating reproducible operational lifetimes. Without TFE, severe dark-spot formation appeared within 1 day of storage (Figure 8d), whereas encapsulated devices maintained uniform emission even after 20 days at ambient conditions. These results confirm that the encapsulation design supports stable operation under bias and provides robust protection against environmental degradation during storage.

## 4. Conclusions

This study presented a stress-balanced, transferable ultrathin platform (~4.5 µm total thickness) based on a urethane-based p(IEM-co-HEMA) polymer superstrate fabricated via iCVD for reliable wearable electronics. The superstrate exhibited high optical transparency, low surface roughness, and strong chemical resistance, providing a stable foundation for inorganic barrier integration. While the integration of ALD Al_2_O_3_/ZnO nano-stratified barriers significantly enhanced the WVTR, tensile residual stress accumulated during ALD growth imposed a critical thickness limit (30 nm), beyond which WVTR stagnated. This limitation is particularly severe in polymer platforms that undergo large curvature because of their thin geometry.

To overcome this challenge, a 40 nm e-beam-deposited SiO_2_ capping layer was integrated with the ALD barrier. This layer introduced compressive residual stress through atomic peening and stabilized the interface via Si–O–Al bond formation. As a result, the critical thickness of the ALD barrier was extended to 60 nm, and the WVTR improved from 2.47 × 10^−4^ to 3.75 × 10^−5^ g/m^2^/day under identical conditions, corresponding to about one order of magnitude enhancement. Importantly, these improvements confirm the feasibility of stress-balancing for ultrathin transferable platforms, and when extended to multilayer encapsulation, this strategy could meet industrial WVTR requirements without excessively thick inorganic films.

OLEDs fabricated on this platform maintained J–V–L characteristics and efficiency roll-off comparable to glass-based references. After water-assisted transfer, the devices showed no additional electrical or optical losses, and following on-skin deformation, they retained LT80 lifetimes of 140–190 h at 400 cd/m^2^ with stable emission for more than 20 days. These results confirm that the proposed stress-balanced encapsulation design is effective not only at the barrier-film level but also at the device level under realistic wearable conditions. By directly linking stress control to device stability, this study bridges materials physics with application-level requirements, establishing a coherent design framework for reliable wearable electronics.

Building on this framework, the employed processes—iCVD, ALD, and e-beam deposition—are notable not only for their effectiveness at the laboratory scale but also for their compatibility with industrial implementation. iCVD enables solvent-free polymer deposition with tunable thickness, ALD provides conformal nano-stratified oxides with high barrier quality, and e-beam deposition offers a straightforward means of stress modulation. Together, these low-temperature and scalable processes suggest a viable route toward large-area manufacturing while maintaining the stress-balanced advantages. Beyond OLEDs, the platform can be extended to OPVs, perovskite photovoltaics, and wearable sensors, underscoring its broad relevance for next-generation skin-mounted and textile-integrated electronics.

## Figures and Tables

**Table 1:** Physical, optical, thermal, and mechanical properties of the p(IEM-co-HEMA) superstrate.

	Property	Measured Value
Surface roughness	Root mean square roughness (R_q_)	1~3 nm
Optical property	Transmittance	91~92%
	Refractive index	1.51
	UV stability	Good
Thermal stability	Thermal decomposition	Onset 250 °C
	Coefficient of thermal expansion (CTE)	50.54 ppm/°C
Mechanical property	Elastic modulus	1~3 GPa
	Yield strength	20~50 MPa
	Yield strain	1.5~3.5%

**Table 2:** Residual stress and average ion energy of SiO_2_ films deposited by different PVD methods.

	Thermal Evaporation	E-Beam Evaporation	Sputtering
Residual stress	389.4 ± 36.6 MPa *	126.5 ± 4.9 MPa	198.9 ± 13.9 MPa
Ion energy	0.255 eV	1.5 eV	22.5 eV

* These are statistical values of average and standard deviation obtained from 5 samples.

**Table 3:** Electrical characteristics of OLEDs fabricated on different platforms measured at 7.5 V.

Measurement Condition	Luminance [cd/m^2^]	Current Density [mA/cm^2^]	Current Efficiency [cd/A]
Glass substrate	946.82 ± 15.2 *	21.89 ± 0.30	4.53 ± 0.15
Polymer platform (before exfoliation)	886.15 ± 12.8	19.29 ± 0.25	4.59 ± 0.14
Polymer platform (after exfoliation)	898.99 ± 14.1	20.18 ± 0.28	4.45 ± 0.13

* These are statistical values of average and standard deviation obtained from 12 samples.

## Data Availability

The original contributions presented in this study are included in the article. Further inquiries can be directed to the corresponding author.

## Abbreviations

The following abbreviations are used in this manuscript:

ALD	Atomic Layer Deposition
Al_2_O_3_	Aluminum Oxide
Alq_3_	Tris(8-hydroxyquinolinato)aluminum
CTE	Coefficient of Thermal Expansion
DEZ	Diethylzinc
EtOH	Ethanol
FT-IR	Fourier Transform Infrared Spectroscopy
HEMA	2-Hydroxyethyl Methacrylate
IEM	Isocyanatoethyl Methacrylate
iCVD	Initiated Chemical Vapor Deposition
IPA	Isopropyl Alcohol
MoO_3_	Molybdenum Trioxide
NPB	N,N′-Bis(naphthalen-1-yl)-N,N′-bis(phenyl)benzidine
OLED	Organic Light-Emitting Diode
OPV	Organic Photovoltaic
PEN	Polyethylene Naphthalate
PET	Polyethylene Terephthalate
PI	Polyimide
PVD	Physical Vapor Deposition
p(IEM-co-HEMA)	Poly(isocyanatoethyl methacrylate-co-2-hydroxyethyl methacrylate)
SiO_2_	Silicon Dioxide
TMA	Trimethylaluminum
WVTR	Water Vapor Transmission Rate
ZnO	Zinc Oxide
